# Functional Brain Imaging in the Clinical Assessment of Consciousness

**DOI:** 10.1371/journal.pbio.1000548

**Published:** 2010-11-30

**Authors:** Michael S. Rafii, James B. Brewer

**Affiliations:** 1 Department of Neurosciences, University of California San Diego, San Diego, California, United States of America; 2 Department of Radiology, University of California San Diego, United States of America; National Institutes of Mental Health, United States of America

## Abstract

Recent findings suggest that functional brain imaging might be used to identify consciousness in patients diagnosed with persistent vegetative state and minimally conscious state. Michael Rafii and James Brewer discuss the potential for fMRI's wider implementation in clinical practice, and associated caveats.

It is 11 p.m. in the hospital and the lone neurology resident has just been called in on a case—a woman had found her 40-year-old husband unresponsive on the floor in their living room, after she heard a loud thud. When she ran to see what happened and saw her husband lying motionless, she immediately called 911. She frantically administered chest compressions and forced breaths until the paramedics arrived and took over resuscitation efforts. After detecting a pulse but almost no blood pressure, they delivered a large bolus of fluids intravenously and inserted an endotracheal tube to ventilate the man's lungs.

In the emergency department, the intake team initiates a workup, including blood electrolytes, arterial blood gases, cardiac enzymes, urine toxicology, cardiac telemetry, and computed tomography brain scan, to determine the cause of the patient's poor responsiveness. With many questions unanswered, the patient is transferred to the intensive care unit and closely monitored.

The distraught family peppers the neurology resident with questions:

Will he make it through this? What are his chances? When will he wake up? Can he understand us? Will he be normal? Is he alive?

In this unfortunate scenario, repeated daily across hospitals around the world, neurologists try to provide the family with an accurate prognosis or, at least, meaningful guidance. In evaluating patients with impaired consciousness, the neurologist assesses the history, the imaging, the laboratory values, and, most importantly, a hands-on examination that interrogates brain function [1]. Ideally, these questions about prognosis and consciousness would be answered in the acute setting, however, it remains difficult to provide answers even after much deliberation over ensuing days, weeks, or even months. Despite best efforts, nearly 40% of consciousness disorders are misdiagnosed [2]. In the setting of such inaccuracy, the possibility of applying new technology toward this problem is clearly enticing. Some have proposed that functional magnetic resonance imaging (fMRI) might allow physicians to “look inside the brain” to identify conscious brain function. Recently, a group of researchers has successfully applied fMRI to identify consciousness in brain-damaged individuals and, in one case, to communicate with the patient.

Here, we discuss the clinical applicability of fMRI for examining disorders of consciousness, and, though we will highlight multiple limitations in its current state that severely restrict clinical application, the approach has yielded results that have gained broader attention from physicians and patient advocates. Future research and methods development are needed before fMRI can be widely applied in the setting of chronically altered consciousness. Even more work is needed before fMRI can be extended to the acute setting portrayed in the scenario above, despite an urgent and pervasive need in clinical neurology for such tools.

Uncertainty in identifying the level of consciousness and in predicting outcome in brain-damaged patients compounds the difficulty for doctors and families as they attempt to make gut-wrenching decisions about the futility of a particular intervention or about withholding further life-sustaining interventions. Neurologists have long sought improved insight into whether or not an individual is conscious and tools are needed for communicating with such individuals. Thus, recent high-profile findings using fMRI have led to widespread interest in near-term application of this technology in the clinical arena, where patients thought to be without consciousness or ability to communicate would be scanned with fMRI to identify brain activation consistent with consciousness. Further, these findings have raised hope that volitional control of brain activation might provide a means by which such patients could communicate their wishes.

Coma (from the Greek “κoµα/koma,” meaning deep sleep) is a profound state of unconsciousness. Coma patients cannot be awakened, fail to respond normally to pain, light, or sound, do not have sleep–wake cycles, and do not take voluntary actions. Coma may result from a variety of conditions, including intoxication, metabolic abnormalities, central nervous system infections, stroke, and hypoxia from cardiac arrest, or from head trauma sustained in falls or car accidents.

Neurologists attempt to quantify the level of coma before deciding on a prognosis. Comas generally last a few days to a few weeks, and rarely more than two to five weeks. Some have lasted several years, after which patients may gradually emerge from the coma, remain in a minimally conscious state [3], progress to a vegetative state [4], or die. Patients in a vegetative state may have awoken from a coma, but presumably have not regained awareness. Patients in a minimally conscious state, unlike those in a vegetative state, exhibit behaviors associated with conscious awareness, although inconsistently. People may emerge from a coma with a combination of physical, intellectual, and psychological impairments. Some patients never progress beyond very basic responses, but many recover full awareness. Recovery usually occurs slowly and gradually.

Predicted chances of recovery are variable owing to different techniques used to measure the extent of neurological damage. All such predictions are based on statistical rates with some level of chance for recovery present. Time is the best general predictor; after four months of coma caused by brain damage, the chance of partial recovery is less than 15%, and the chance of full recovery is even less.

Neurologists routinely use the Levy criteria [1] to help prognosticate, with percentage of recovery assigned to different responses on neurological testing over the first few days post-injury. In this landmark study, presence or absence of pupillary response to light, blink response to corneal stimulation, and motor response to pain recorded across the first days of coma were linked to outcome percentages in 310 patents with anoxic brain injury studied over one year. No similar study exists on such a scale, and so these outcome numbers represent the neurologists' best guess for outcome in the days following a patient's anoxic brain injury. But without similar data informing prognosis of other forms of brain injury such as traumatic brain injury, the Levy criteria are often inappropriately extended to prognosticate in these other cases.

Technology has been used toassist in assessing the health of the brain and its likelihood of recovery in these situations. Structural brain imaging can help identify whether profound brain damage is present, such as that resulting from a large intracranial hemorrhage, infarction, or tumor. Diffuse brain edema might suggest that neural damage is extensive and recovery is likely to be poor [5]. However, every case is unique, and most neurologists have stories of patients whose level of recovery was astounding despite evidence of widespread brain damage on structural imaging.

Electroencephalography may complement structural imaging, as it allows some exploration of the patient's brain function. By examining the injury's disruption of brain electrical rhythms, the physician can gain further information about the extent of brain injury and likelihood of patient consciousness, but assessment of brain rhythms does not greatly improve prognostic accuracy, nor does it directly assess consciousness. Electroencephalographic evidence of brain response to sensory nerve stimulation does appear to improve prognostic accuracy [6]. Specifically, bilateral absence of cortical evoked potentials reliably predicts unfavorable outcome in comatose patients after cardiac arrest [7], but it, too, says little about the presence or absence of consciousness.

Recently, a paper published in *The New England Journal of Medicine* [8] reported that imaging technology revealed conscious intent in patients with brain injury. Using fMRI, they showed evidence of willful brain activation in five patients (out of 54 studied) in vegetative or minimally conscious states. Further, one patient with a diagnosis of vegetative state was able to correctly answer yes or no questions by activating different areas of his brain through visualization of different activities while he was undergoing fMRI, despite being unable to show any signs of consciousness at the bedside.

Understandably, this report has gained much attention from physicians and families caring for patients presumed to be minimally conscious. Physicians at the present authors' institution, including the present authors themselves, have been approached by families to perform the test as part of the evaluation of patients with impaired consciousness from various etiologies. As such, it is worthwhile noting that all five patients in the reported study who were in a vegetative or minimally conscious state and were found to be responsive on fMRI, had sustained traumatic brain injury rather than anoxic brain injury. This does not necessarily serve as an effective argument against testing, however, when the family is desperate for an answer about their loved one.

How can one argue against looking for subclinical signs of consciousness in patients when a technology for doing so exists? No doubt- the idea is tantalizing, and the authors are to be lauded for taking on such a herculean task of performing fMRI in this oft-neglected patient population. Indeed, the challenges of performing fMRI in patients are many and varied. But the enthusiasm for wider application of the authors' methodology in the clinical realm must be tempered, particularly when considering the limitations of the technology and the potential for misinterpretation of results. The authors were not measuring neural activity associated with consciousness per se, but rather measuring neural activity unlikely to be generated in the setting of unconsciousness. In the end, the neural activity was analogous to a lever the patient could pull to communicate, and, if the patient was not conscious, the brain region would not activate; the lever would not be pulled.

As with any test, the issues surrounding false positives and false negatives are important, but these issues are particularly salient when considering wider use of this procedure. Errors in either direction could be harmful. An unconscious patient misclassified through fMRI as being conscious might be subjected to inappropriately aggressive or prolonged treatment. But what about a conscious patient misclassified as unconscious? Going forward, it seems that a treating physician should assume that some level of consciousness could exist, regardless of test outcome. This is especially so in light of evidence for fluctuations of consciousness, even in the minimally conscious state. Such evidence demands that negative test results would be interpreted with great caution, especially before allowing it to influence care in any way.

Though successful in this study and perhaps the best of all current options, it is hard to argue that fMRI is a technology well-suited for this population. Even under ideal circumstances, it is an extremely poor communication device. The signal is highly dependent on subject cooperation, as artifacts due to subject motion disrupt the images themselves, and subtle misunderstanding of task instructions can lead to uninterpretable or misinterpretable data. In this population, presentation of auditory stimuli is fraught with additional challenges. Unlike auditory fMRI studies in the healthy population, which have been performed successfully despite the noisy scanning environment, here there is no possibility of titrating sound level for the individual and confirming that the stimuli are audible to the patient.

Furthermore, the data must be reliable in the individual patient. Most fMRI experiments must pool activity across a dozen or more subjects to obtain reliable signal; however, the authors laid the foundation for this study through extensive prior work that showed the validity of their approach in individuals [9]. Most of that validation was performed in healthy subjects with normal brain anatomy and function, and it remains unclear whether one can count on such reliability when the technique is exported to centers without the specific expertise of the study's authors. Optimal statistical thresholds for such studies also require further study. The present study selected the *p*<0.05 level with accepted correction for statistical comparisons across the whole brain. Individual fMRI data is inherently noisy and is especially so for tasks involving higher-level cognitive function, as opposed to primary sensory function. Scanners outside the authors' institutions will vary in the signal-to-noise characteristics. The authors' validation studies, therefore, might be less relevant to exporting the technique to other centers, as the same selected statistical threshold may yield additional spurious activations that have a chance of falling within the targeted region of interest.

Nevertheless, the study demonstrates the potential of using fMRI tasks, and resulting brain activation, as an approach to communicate with patients otherwise unable to make their needs known. It represents a creative use of the localized activity pattern yielded by fMRI, combined with reverse inference, to gauge the processes taking place in the brain of a patient. It is hard to deny that the findings are indeed important. Excitement is usually the emotion that accompanies a new and important finding, but in this case, one is dismayed that patients presumed to be with minimal consciousness might instead meet a new definition of the “locked-in syndrome”—conscious but unable to communicate even with simple saccades or subtle blinks.

In fact, one would hope that the authors were mistaken in concluding that no alternative means of communication were possible for the one responsive patient. After all, one can never fully explore the universe of behavioral communication methods. Yet, the expertise of the authors in this field suggests that the prior testing was extensive, and, as such, the data support a relatively dismal notion—that cases exist where the *only* means of detecting consciousness is by looking inside the brain. Until now, all such patients have missed detection. These well-characterized individual patients, and any others subsequently discovered, would prove highly valuable for developing novel techniques for binary means of communication beyond fMRI, which could be used in MCS, PVS, and coma. The authors no doubt exhausted the accepted approaches used in clinical neurology that explore for subtle eye, tongue, or finger movements. Might physicians and researchers with expertise in using other biomarkers, such as galvanic skin response, electrodiagnostic approaches, or a host of other procedures also find success in communicating with such patients? Perhaps. Or perhaps all other techniques outside of fMRI would fail, but it seems important to test simpler techniques against the new fMRI procedure that presently is the gold standard in these rare cases.

It is difficult to identify more than a few direct health benefits of the many millions of dollars invested by governments in fMRI research, and it is increasingly recognized that fMRI yields only a partial picture of brain function. In fact, cognition and consciousness are more distributed processes than can be revealed by blobs on an fMRI activation map. Advances are needed before promoting the widespread use of such technology to answer clinical questions of coma and consciousness. Developing robustness to motion and other artifacts while improving signal-to-noise for use in individual patients are clear targets, but conceptual advancements are also needed. The current focus on localizing regional activity that correlates with an isolated cognitive function will likely be insufficient, and, the field may benefit from more recent focus on putative functional networks revealed though inter-regionally correlated activity. Such activity can be revealed even in the absence of a task [10], which would further improve the applicability in patient populations poorly able to follow task instructions, including the minimally conscious [11]. It remains to be seen, however, whether these networks can be reliably associated with levels of brain function or presumed consciousness, or even, perhaps, if they are simply an epiphenomenon of thalamocortical rhythms better characterized through electroencephalography. Nonetheless, fMRI remains an extremely powerful tool for exploring human brain function, as it is able to reveal changes in regional blood flow in response to regional neural activity and does give “a look inside the brain,” albeit a limited one [12]. Advances are expected to further improve clinical applicability to individual patients. Given such advances and despite the myriad challenges to consistent application in the clinical setting, fMRI using blood oxygen level–dependent responses or other developing approaches [13] might eventually serve to help answer some of the most difficult questions doctors and families face when clinically evaluating brain function and prognosis in the minimally conscious patient.

## Abbreviations

fMRI: functional magnetic resonance imaging
